# Oncogene withdrawal engages the immune system to induce sustained cancer regression

**DOI:** 10.1186/2051-1426-2-24

**Published:** 2014-07-15

**Authors:** Stephanie C Casey, Yulin Li, Alice C Fan, Dean W Felsher

**Affiliations:** 1 Division of Oncology, Departments of Medicine and Pathology, Stanford University School of Medicine, 269 Campus Drive, CCSR 1105, Stanford 94305-5151, CA, USA

**Keywords:** Oncogene addiction, MYC, Tumor microenvironment, Tumor immunology

## Abstract

The targeted inactivation of a single oncogene can induce dramatic tumor regression, suggesting that cancers are “oncogene addicted.” Tumor regression following oncogene inactivation has been thought to be a consequence of restoration of normal physiological programs that induce proliferative arrest, apoptosis, differentiation, and cellular senescence. However, recent observations illustrate that oncogene addiction is highly dependent upon the host immune cells. In particular, CD4^+^ helper T cells were shown to be essential to the mechanism by which MYC or BCR-ABL inactivation elicits “oncogene withdrawal.” Hence, immune mediators contribute in multiple ways to the pathogenesis, prevention, and treatment of cancer, including mechanisms of tumor initiation, progression, and surveillance, but also oncogene inactivation-mediated tumor regression. Data from both the bench and the bedside illustrates that the inactivation of a driver oncogene can induce activation of the immune system that appears to be essential for sustained tumor regression.

## Introduction

### Capitalizing on oncogene addiction: a therapeutic objective

**Figure 1:**
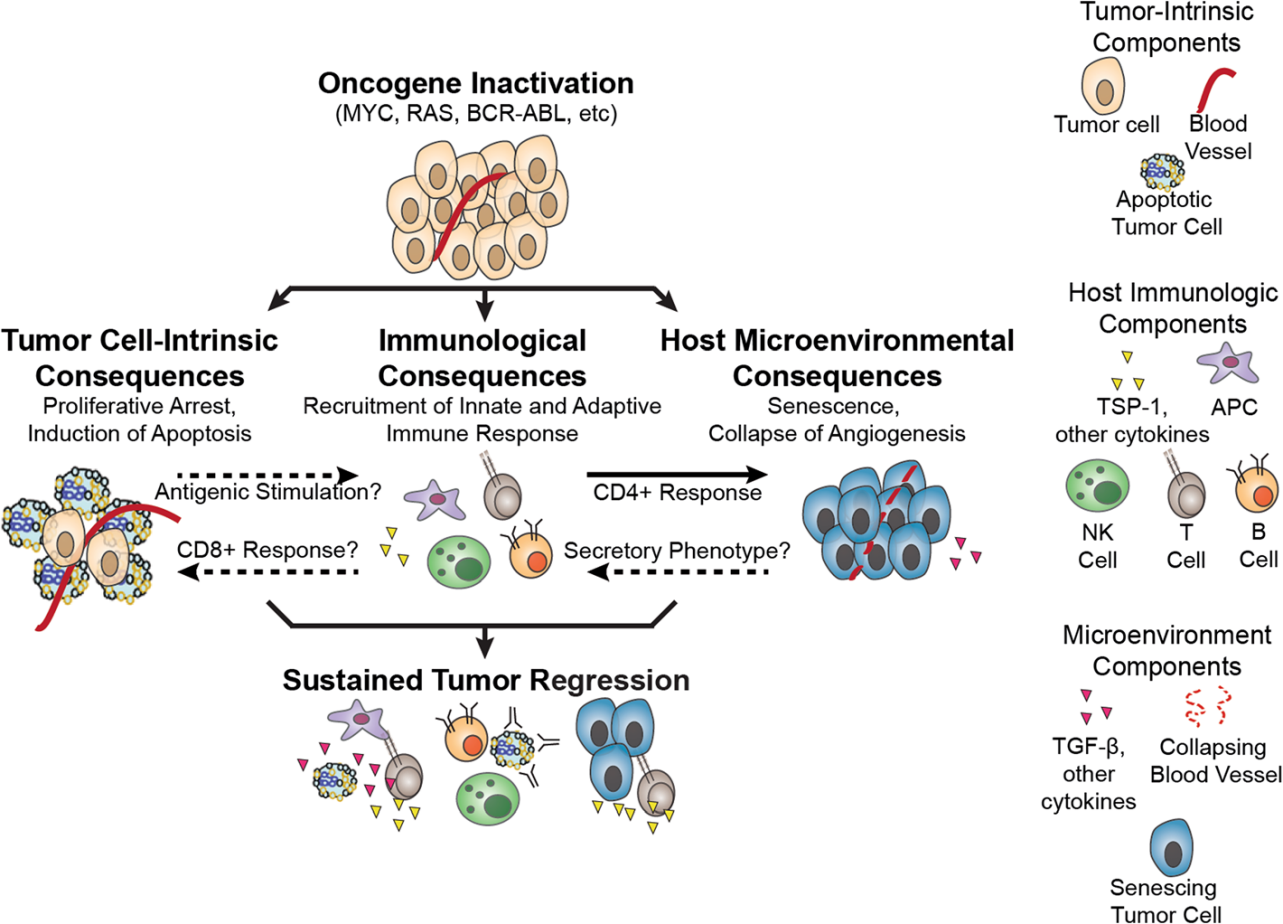
**The host immune system is required for sustained tumor regression following oncogene withdrawal.** Following oncogene inactivation in a mouse model by transgenic methods or in patients by oncogene-targeted therapy, there are tumor cell-intrinsic consequences, immunological consequences, and host microenvironmental consequences. Tumor cell-intrinsic consequences include proliferative arrest and the induction of apoptosis. Dying tumor cells and antigen debris may stimulate an immune response, which may in turn feed back in to the tumor cell-intrinsic consequences. The immune response, particularly helper T cells, can influence environmental consequences, including the induction of senescence and the collapse of angiogenesis. Lastly, senescing tumor cells may have a secretory phenotype, which in turn may influence the immune system. Taken together, these three components lead to a remodeling of the entire tumor (both in the cancer cells and in the environment) and contribute to lasting tumor regression and protection from relapse.

The inactivation of a single oncogene can result in the dramatic and sustained regression of some cancers [1-4]. Targeted inactivation of an oncogene can be associated with proliferative arrest, apoptosis and/or senescence, and differentiation [3]. Oncogene addiction appears to be a consequence of the restoration of physiological programs [2,5], but also has been described as a consequence of synthetic lethality [6] and the differential decay of survival and apoptosis programs [7]. “Oncogene withdrawal” occurs upon suppression of initiating genetic events in tumors [8,9]. It is not known when a cancer will be addicted to a particular oncogene [4]. Oncogene addiction has been thought to occur through host cell autonomous, tumor intrinsic mechanisms. Yet, recent observations illustrate that oncogene addiction has both cell autonomous as well as immune-mediated mechanisms [10-14] (Figure 1).

Oncogene addiction has been largely studied in mouse models. However, tumor regression following oncogene inactivation has been observed in response to targeted therapeutics in humans including molecules that target BCR-ABL or c-Kit, EGFR, ALK, BRAF V600E, PML-RARα, and HER2/neu for the treatment of leukemia, lung adenocarcinoma, non-small cell lung cancer, melanoma, and breast cancer [15-23]. Other drugs are in clinical investigation, including those that target JAK2, MDM2, and PI3 Kinase [24-31]; drugs that target RAS [32] and MYC [33,34] are in early development. It remains to be seen if these agents specifically take advantage of oncogene addiction. In general, little has been done to study the mechanism of action of these therapeutic agents in human patients.

Experimental mouse models have been a particularly tractable approach to interrogating the mechanism of oncogene addiction. Transgenic mouse models employing strategies that enable the conditional expression of oncogenes have been used to illustrate that cancers initiated by an oncogene, such as MYC, RAS, BCR-ABL, MET, and BRAF, are reversible upon suppression of the oncogene [1,35-40].

## Review

### Cancer and the immune system: a complex relationship

The mechanisms by which targeted therapies engage oncogene addiction have been presumed to be cell autonomous. However, oncogene inactivation causes dramatic changes in the microenvironment including the shut down of angiogenesis [41-44] and the recruitment of host effector cells, including innate and adaptive immune cells [4,45-48]. Thus it appeared likely that the immune system is playing an active role in the mechanism of tumor regression following oncogene inactivation.

Furthermore, it is well known that the immune system is a barrier to tumorigenesis [49]. Hosts with absent or suppressed immune systems have a greatly increased incidence of many different types of cancer [50,51]. Patients who are transplant recipients and take drugs that suppress their adaptive immunity demonstrate dramatically increased incidences of lymphoma and squamous cell carcinoma [52-54]. Moreover, patients who are immunosuppressed also have an impeded response to cancer therapy with a decreased overall and progression-free survival [55,56]. Thus, immune surveillance mechanisms are critical both to the prevention as well the efficacy of conventional treatment of these cancers [57-59] and are a critical component to the therapeutic efficacy of agents for cancer [54,60-62].

Correspondingly, the activation of the immune system through specific immune-based therapies is efficacious for the treatment of some cancers. This includes antibodies that target cancer cells, such as Rituximab [63] and Trastuzumab [64], as well as antibodies or drugs that modulate immunostimulatory or immunoinhibitory signals [65-67], such as anti-CTLA-4 [68] and anti-PD-L1 [69]. The combination of conventional chemotherapy with targeted immune therapy has emerged as an effective approach for the treatment of some cancers.

### Oncogene inactivation activates the immune system

Recent studies in experimental mouse models illustrate the mechanisms by which oncogene “withdrawal” results in immune activation (Figure 1, [24,45]). In a tetracycline-regulated conditional mouse model of MYC-induced T cell Acute Lymphoblastic Leukemia (T-ALL), the tumor cells undergo proliferative arrest and death within 2 days of turning off the MYC oncogene via tumor intrinsic, host independent, immune independent mechanisms. Subsequently, between 2 and 5 days, there is a recruitment of immune effector cells that are required to induce cellular senescence of tumor cells and the shut down of angiogenesis in the tumor microenvironment [70]. The kinetics of tumor regression, the extent of tumor regression, and the ability to maintain sustained tumor regression are all compromised in immunodeficient hosts.

Provocatively, CD4^+^ helper T cells were found to be the key immune effector required for oncogene inactivation-induced tumor regression in the conditional MYC-driven T-ALL mouse model. The CD4^+^ T cells are likely to contribute to tumor regression through many mechanisms. Of note, CD4^+^ T cells can express a variety of cytokines that have been implicated in the regulation of cellular senescence and/or angiogenesis [71-74]. CD4^+^ T cells may also be working via direct cellular interactions with the tumor cells or host stromal cells in the tumor microenvironment. Finally, CD4^+^ T cells appear to recruit other immune and host cells.

The CD4^+^ helper T cells must express thrombospondins in order to contribute to tumor regression following oncogene inactivation [45]. TSP-1 has been suggested to be a key regulator of both angiogenesis and senescence [75]. Moreover, CD47, the receptor of TSP-1, is a key regulator of the immune response [76]. TSP-1 and CD47 have been suggested to regulate cellular senescence [75,77,78]. However, there is also a general induction of anti-tumor and a suppression of pro-tumor cytokines after oncogene inactivation that occurs only in immunocompetent hosts [45]. Hence, specific secreted factors are likely to contribute to the mechanism of oncogene addiction and withdrawal.

How oncogene inactivation recruits a response of CD4^+^ T cells is not known. There are several possibilities. First, oncogenes such as MYC have been suggested to regulate the expression of molecules that may be immunosuppressive and/or regulate angiogenesis. Hence, MYC inactivation could lead to the direct change in expression of cytokines by tumor cells, thereby recruiting immune cells [79]. Second, oncogene inactivation could activate an immune response through immunogenic cell death that in turns activates the immune response [80]. Identifying the specific mechanism of the immune activation and response could suggest important strategies for monitoring and implementing a therapeutic response [10].

Importantly, many other immune effectors are likely to contribute to the response of targeted therapies. This is potentially governed by the unique genetic and cellular context of each tumor [81,82]. In other mouse models, investigators have noted that innate immune cells such as mast cells [83], macrophages [84], and other antigen-presenting cells (APCs) may function as barriers to tumor growth and facilitators of tumor regression. Thus, it is likely that these other innate and adaptive immune cells contribute to the mechanism of oncogene addiction and tumor regression following oncogene inactivation.

### In the clinic: targeted oncogene inactivation and immune response

Oncogene addiction has been studied in a more limited manner in human patients. Some studies indicate that the host immune response is essential for the optimal response to conventional chemotherapy and radiotherapy [85-87]. A major potential limitation of conventional therapeutics is that they often suppress the immune response [88].

Other correlative studies suggest that an immune response may contribute to the mechanism of targeted oncogene inactivation. In human patients with BCR-ABL^+^ gastrointestinal stromal tumors (GIST) treated with Imatinib, IFN-γ secretion by NK cells in the peripheral blood is associated with a better clinical response [89]. Similarly, the inhibition of BRAF both directly inhibits tumor growth but also appears to activate the immune system [90]. The combination of a BRAF inhibitor, Vemurafenib, with immune therapy may be more effective in the treatment of tumors [91]. Moreover, Vemurafenib was associated with intratumoral accumulation of adoptively transferred T cells [92] as well as increased intratumoral numbers of CD4^+^ or CD8^+^ T cells [90] and this was associated with a better prognosis [90]. Other studies have shown that BRAF inhibition is associated with the reduction of immunosuppressive cytokines and chemokines [93]. Ongoing clinical studies are examining if Vemurafenib in combination with immunotherapy is more clinically effective [94,95].

Other targeted therapies may induce an immune response in addition to their tumor-specific effects. Sunitinib, which targets PDGFR, RET, and KIT, recruits an immune response that may contribute to its mechanism [96] through the induction of IFN-γ-producing T cells [97] and decreased regulatory T cells [97,98]. Arsenic and all-trans-retinoic-acid (ATRA), used for the treatment of PML-RARα acute promylecytic leukemia, is associated with altered antigen presentation [99]. Bortezomib is a proteasome inhibitor used in the treatment of hematopoietic tumors and is associated with the recruitment to tumor sites of CD8^+^ T cells and dendritic cells [100]. The EGFR inhibitor, Erlotinib, is effective in the treatment of non-small cell lung cancer and is associated with increased intratumoral numbers of dendritic cells [101]. Trastuzumab targets HER2/neu for the treatment of breast and ovarian cancer and may require an NK cell response [102,103]. Thus, targeted inhibition of oncogenes may be efficacious in part through the activation of an immune response.

In some cases, targeted inactivation of oncogenes could inhibit an immune response and impede the efficacy of an anti-tumor therapeutic. For example, inhibition of MAPK/extracellular signal-regulated kinase kinase (MEK) results in T cell inhibition [104]. Imatinib can affect the immune response in a multitude of ways [24,45,105-109]. Thus, it will be pivotal to consider how targeted oncogene inactivation can induce or suppress an immune response and how this may contribute to the mechanism of action of anti-neoplastic agents.

### Therapeutic implications for oncogene-targeted therapies

Experimental evidence and clinical observations suggest that targeted oncogene inactivation generates an anti-tumor immune response. More generally this suggests that targeted oncogene inactivation can be exploited as an immune therapy. Unlike conventional chemotherapy or radiotherapy, the judicious choice of agents that target specific oncogenes may lead to tumor regression both by directly targeting tumor cells and indirectly by inducing a robust immune response. If this were the case, it would have several practical implications for the development and application of therapeutics.

First, the combination of oncogene-targeted therapy with specific immunomodulatory therapy may further increase the clinical response and long-term survival of patients [94,110,111]. Pointedly, immune activation may be essential to prevent the emergence of therapy-resistant tumor cells, which can lead to tumor recurrence [112,113]. Hence, the identification of the best agents to prompt oncogene withdrawal will require examination of the efficacy of these therapies with consideration of their ability to induce both cell autonomous and host-dependent mechanisms of tumor regression.

Several targeted therapies are currently approved or under investigation in combination with immunomodulatory therapies (Table 1). For the treatment of melanoma, MEK and VEGF inhibitors are being administered with Ipilimumab [114,115] and IL-2 [116], respectively. BRAF inhibitors are being examined together with Ipilimumab [117]. Ipilimumab is also being interrogated in combination with Brentuximab for the treatment of Hodgkin’s Lymphoma [118] and with Crizotinib for non-small cell lung cancer [119]. Ipilimumab and anti-PD-L1 inhibitors are being analyzed in combination with Erlotinib in non-small cell lung cancer [119,120].

Targeted therapies together with immune-based therapies are also being examined for the treatment of other types of cancer. Lenalidomide and Bortezomib are being examined for treatment of multiple myeloma [121], Lenalidomide and Ibrutinib are under investigation for Chronic Lymphocytic Leukemia [122], and Nivolumab is being administered with Sunitinib for renal cell cancer [123]. Additionally, the mTOR inhibitor Temsirolimus is being studied with Interferon-α for renal cancer [124]. Imatinib and Rituximab are being investigated in combination with Nivolumab [125] or Pidilizumab [126]. Trastuzumab is under investigation with peptide vaccines and cytokines [127]. These investigations may identify combinations of targeted and immune-based therapies that are more efficacious for the treatment of cancer. Furthermore, the appreciation that immune activation may be a critical component to the efficacy of therapeutics may be important for the measurement and maximization of their clinical efficacy.

**Table 1:** Targeted therapies studied or under investigation in cooperation with immune therapies

**Target protein(s)**	**Tumor type**	**Targeted therapy**	**Immune therapy**	**Refs**
*ALK*	Non-Small Cell Lung Cancer	Crizotinib	Ipilimumab	[119]
*BCR-ABL*	CML, GIST	Imatinib, Dasatinib	Interferon, Nivolumab	[125]
*BRAF*	Melanoma	Vemurafenib, Dabrafenib	Ipilimumab	[117]
*BTK*	Chronic Lymphocytic Leukemia	Ibrutinib	Lenalidomide	[122]
*CD20*	Follicular Lymphoma	Rituxamab	Pidilizumab	[126]
*CD30*	Hodgkin’s Lymphoma	Brentuximab	Ipilimumab	[118]
*EGFR*	Non-Small Cell Lung Cancer	Erlotinib	Ipilimumab, anti-PDL1 (MPDL3280A)	[119,120]
*HER2/neu*	Breast Cancer	Trastuzumab	E75 peptide + GM-CSF	[127]
*MEK*	Melanoma	Trametinib	Ipilimumab	[114,115]
*mTOR*	Renal Cell Cancer	Temsirolimus	Interferon-α	[124]
*PDGFR, RET, or KIT*	Kidney Cancer	Sunitinib	Nivolumab	[123]
*Proteosome, NF-kB*	Multiple Myeloma	Bortezomib	Lenalidomide	[121]
*VEGF*	Melanoma	Aflibercept	IL-2	[116]

## Conclusions

Experimental and clinical observations suggest a model of oncogene addiction and a role for the immune system (Figure 1). The inactivation of an oncogene in a tumor appears to initiate cancer cell-intrinsic programs of tumor regression including proliferative arrest, differentiation, and apoptosis, as well as immune-dependent modulation of the microenvironment that contributes to cellular senescence and the shut down of angiogenesis. These mechanisms are collectively required for complete and sustained tumor regression.

Oncogene inactivation in a tumor results in activation of an immune response (Figure 1). The mechanisms by which this occurs are not defined. These mechanisms potentially involve both direct mechanisms related to the production of immune recruiting cytokines as well as more indirect mechanisms such as immunogenic cell death. Many cellular and cytokine effectors are likely to be involved, including CD4^+^ T cells, CD8^+^ T cells, B cells, and innate immune cells such as macrophages and NK cells (Figure 1). It is possible that the impairment of specific cellular, humoral, or chemokine mechanisms would facilitate the re-emergence of tumor cells that are refractory to targeted therapy.

There are several practical implications of this model. First, successful targeted therapy against a cancer is likely to require an intact host immune system. Second, the measurement of the efficacy of a targeted therapy is likely to be most readily defined through interrogation of immune activation after drug administration. Third, the early development of therapeutic agents should be performed using model systems that have an intact host immune system as opposed to in vitro model systems or xenograft model systems in severely immunocompromised animals.

Our model predicts that the immune system not only directly eliminates tumor cells but also plays a critical role in modulating the tumor microenvironment. Diagnostic assays that detect an immune response may predict the therapeutic efficacy of oncogene-targeted agents. Strategies need to be developed that would enable the measurement of these effector cells and molecules before and after therapeutic treatment. This could include in situ measurements in patients using flow cytometry analysis of immune effector cells, proteomic and genomic analysis, and noninvasive molecular imaging methods.

Finally, the most effective clinical strategy to treat tumors will likely require a coordination of therapies that target oncogenes in combination with the activation of specific immune effectors. Conversely, existing conventional chemotherapies that often impede an immune response may antagonize the efficacy of targeted therapeutics. Hence, mechanistic insight into how oncogene withdrawal prompts immune activation may actualize rationale therapeutic strategies.

## Competing interests

The authors declare that they have no competing interests.

## Authors’ contributions

SCC and DWF conceived of and wrote the review. YL helped write the review. ACF provided a clinical perspective on targeted and immunological therapies. All authors read and approved the final manuscript.
